# HCIV-1 and Other Tailless Icosahedral Internal Membrane-Containing Viruses of the Family *Sphaerolipoviridae*

**DOI:** 10.3390/v9020032

**Published:** 2017-02-18

**Authors:** Tatiana A. Demina, Maija K. Pietilä, Julija Svirskaitė, Janne J. Ravantti, Nina S. Atanasova, Dennis H. Bamford, Hanna M. Oksanen

**Affiliations:** 1 Department of Biosciences and Institute of Biotechnology, Viikinkaari 9, FIN-00014, University of Helsinki, Helsinki, Finland; tatiana.demina@helsinki.fi (T.A.D.); julija.svirskaite@helsinki.fi (J.S.); janne.ravantti@helsinki.fi (J.J.R.); nina.atanasova@helsinki.fi (N.S.A.); dennis.bamford@helsinki.fi (D.H.B.); 2 Department of Food and Environmental Sciences, Viikinkaari 9, FIN-00014, University of Helsinki, Helsinki, Finland; maija.pietila@helsinki.fi

**Keywords:** *Sphaerolipoviridae*, *Alphasphaerolipovirus*, halovirus, archaeal virus, tailless icosahedral virus, membrane virus, halophilic archaea, virus classification, International Committee on Taxonomy of Viruses, structure-based viral lineage

## Abstract

Members of the virus family *Sphaerolipoviridae* include both archaeal viruses and bacteriophages that possess a tailless icosahedral capsid with an internal membrane. The genera *Alpha-* and *Betasphaerolipovirus* comprise viruses that infect halophilic euryarchaea, whereas viruses of thermophilic *Thermus* bacteria belong to the genus *Gammasphaerolipovirus*. Both sequence-based and structural clustering of the major capsid proteins and ATPases of sphaerolipoviruses yield three distinct clades corresponding to these three genera. Conserved virion architectural principles observed in sphaerolipoviruses suggest that these viruses belong to the PRD1-adenovirus structural lineage. Here we focus on archaeal alphasphaerolipoviruses and their related putative proviruses. The highest sequence similarities among alphasphaerolipoviruses are observed in the core structural elements of their virions: the two major capsid proteins, the major membrane protein, and a putative packaging ATPase. A recently described tailless icosahedral haloarchaeal virus, *Haloarcula californiae* icosahedral virus 1 (HCIV-1), has a double-stranded DNA genome and an internal membrane lining the capsid. HCIV-1 shares significant similarities with the other tailless icosahedral internal membrane-containing haloarchaeal viruses of the family *Sphaerolipoviridae*. The proposal to include a new virus species, *Haloarcula virus HCIV1*, into the genus *Alphasphaerolipovirus* was submitted to the International Committee on Taxonomy of Viruses (ICTV) in 2016.

## 1. Introduction

Viruses associated with the domain *Archaea* are known to infect crenarchaeal hyperthermophiles, as well as euryarchaeal halophiles, thermophiles, and methanogens. The total number of isolates is currently close to 150, and includes ~80 with sequenced genomes [1,2,3]. So far, all of the characterized archaeal viruses have a DNA genome, which can be either linear or circular double-stranded DNA (dsDNA) molecule or a circular single-stranded DNA (ssDNA) [2,3]. In addition, numerous putative provirus sequences have been detected in the genomes or plasmids of various archaeal strains [4,5,6,7]. Metagenomics studies have indicated the presence of archaeal RNA viruses, but none have been isolated to date [8].

Collectively, archaeal viruses display 14 morphotypes, some of which are unique to this group [2,3,9,10]. Archaeal virus morphotypes include tailless icosahedral viruses, of which only seven have been isolated and described. Two of these, the *Sulfolobus* turreted icosahedral viruses STIV and STIV2, infect hyperthermoacidophilic crenarchaea [11,12], while the other five infect halophilic euryarchaea (see below). STIV, the first of these to be characterized, remains one of the best studied archaeal viruses [12,13,14,15,16,17]. Both STIV and STIV2 have been classified as members of the genus *Alphaturrivirus*, the sole genus in the family *Turriviridae*. An amazing feature of the STIV life cycle is the mechanism of viral egress through pyramid-like structures formed on the surface of its *Sulfolobus solfataricus* hosts [18,19]. STIV's 74 nm diameter icosahedral capsid encloses an internal membrane and bears a turret-like appendage at each vertex [12]. Packaged within is a circular dsDNA genome of 17.3 kb [12].

The STIV major capsid protein (MCP) possesses a double β-barrel fold and is arranged into trimeric capsomers of pseudo-hexagonal symmetry (triangulation number *T* = 31 dextro) [12,15,20]. Archaeal virus STIV2, which infects *Sulfolobus islandicus*, is closely related to STIV [11]. Sequence alignment and homology modelling suggested that the STIV2 MCP also adopts a double β-barrel fold [11]. Similarly folded trimeric capsomers with pseudo-hexameric bases are also found in tailless icosahedral dsDNA viruses associated with bacteria and eukaryotes, e.g., bacteriophages PRD1 and PM2 [21,22], human adenovirus [23], and algal *Paramecium bursaria* Chlorella virus 1 (PBCV-1) [24]. Moreover, these double β-barrel folds are also present in vaccinia virus [25] and in the virophage Sputnik [26]. Based on their conserved virion architecture and MCP fold, all of these viruses have been grouped into the PRD1-adenovirus structural lineage [27,28,29,30,31], a lineage that also includes the algal virus PpV01 [32], Chilo iridescent virus (CIV) [33], Mimivirus, and African swine fever virus [27]. Additional viral structural lineages have been established or suggested that also unite viruses that infect hosts from all three domains of cellular life [30,34].

The other five of the seven archaeal tailless icosahedral viruses infect halophilic euryarchaeal strains. Four of them, SH1 [35,36,37], *Haloarcula hispanica* icosahedral virus 2 (HHIV-2) [38,39], SNJ1 [40], and PH1 [41], were classified in the family *Sphaerolipoviridae* together with viruses of thermophilic bacteria. The recently described *Haloarcula californiae* icosahedral virus 1 (HCIV-1) [42] has been proposed to belong to the same family. Here, we provide an overview of the *Sphaerolipoviridae* family with an emphasis on the genus *Alphasphaerolipovirus*.

## 2. Family *Sphaerolipoviridae*

The family *Sphaerolipoviridae* was approved by the International Committee on Taxonomy of Viruses (ICTV) [43] in 2014 [44]. The family name describes the spherical appearance of the membrane-containing virions, i.e., the Latin *sphaero* for “sphere” and the Greek *lipos* for “fat”. The family contains three genera: *Alpha*-, *Beta*-, and *Gammasphaerolipovirus*, for which the type species are *Haloarcula hispanica virus SH1*, *Natrinema virus SNJ1*, and *Thermus virus P23-77*, respectively (Table 1). The *Alpha*- and *Betasphaerolipovirus* genera comprise archaeal viruses, while bacteriophages P23-77 and IN93 belong to the genus *Gammasphaerolipovirus* [44,45,46,47,48,49]. Bacteriophages P23-72 and P23-65H that infect *Thermus thermophilus* might also be members of that genus [44]; their structural protein profiles are highly similar to that of P23-77 and they originated from the same hot spring [45,50]. The genus *Betasphaerolipovirus* currently contains only one species, *Natrinema virus SNJ1* [7,40,44]. The genus *Alphasphaerolipovirus* comprises three virus species: *Haloarcula hispanica virus SH1*, *Haloarcula hispanica virus PH1*, and *Haloarcula hispanica virus HHIV2* [35,36,37,38,39,41,51]. Recently, we isolated and characterized the tailless icosahedral archaeal virus HCIV-1 and showed that HCIV-1 is closely related to SH1, PH1, and HHIV-2 [42,52]. In 2016, we submitted a proposal to the Executive Committee (EC) of the ICTV to add HCIV-1 as a new virus species, *Haloarcula* virus HCIV1, in the genus *Alphasphaerolipovirus* of the family *Sphaerolipoviridae*. The taxonomic status of HCIV-1 was approved by the EC and now awaits ICTV ratification.

**Figure 1:**
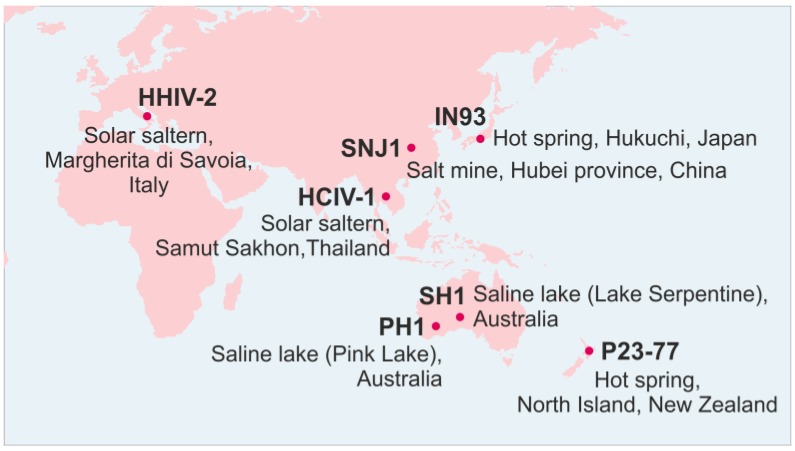
Sphaerolipovirus isolation locations. Map source: Freepic [54].

Sphaerolipoviruses with a lytic infection cycle have been isolated from various locations by plaque assay (Figure 1) [37,41,45,50,52,53]. Induction of lysogenic host strains has yielded two temperate virus isolates: bacteriophage IN93 and archaeal virus SNJ1 [40,47].

Bacterial and archaeal sphaerolipoviruses share a similar virion architecture: an icosahedral capsid enclosing an internal membrane [7,35,36,37,38,39,40,41,42,45,46,48]. The icosahedral virions are ~80 nm in diameter, except for PH1 (~50 nm) and IN93 (~130 nm); their genomes are either linear or circular dsDNA molecules and range from 16,341 bp for SNJ1 to 31,314 bp for HCIV-1 (Table 1).

The three members of the genus *Alphasphaerolipovirus* (SH1, HHIV-2, and PH1) have highly similar structural protein profiles, and the recently isolated HCIV-1 follows the same pattern (Table 1) [35,37,38,41,42]. In addition, the linear dsDNA genome of HCIV-1 is similar in length to those of SH1, HHIV-2, and PH1, and all four genomes are collinear. It is possible to obtain high titer virus stocks for all of them, which makes them suitable model systems for halovirus research. Here, we present the three current members of the genus *Alphasphaerolipovirus* and the newly proposed member, HCIV-1, and compare the available information on their life cycles, genomics, and virion components. We also review the related proviruses that have been identified in the genomes of haloarchaeal strains.

## 3. Life Cycles of Viruses in the Genus *Alphasphaerolipovirus*

SH1, which was isolated from Lake Serpentine in Australia [37], is, so far, the best characterized sphaerolipovirus [35,36,37,51,55,56]. PH1 originates from another saline lake in Australia (the Pink Lake), whereas HCIV-1 and HHIV-2 were isolated from solar salterns (Table 1, Figure 1). The host range of sphaerolipoviruses includes typically 2‒4 hosts each [7,37,41,45,48,50,52,53]. Alphasphaerolipoviruses SH1, HHIV-2, PH1, and HCIV-1 each infect up to four different haloarchaeal strains belonging to the genera *Haloarcula* or *Halorubrum*. All alphasphaerolipoviruses are able to infect *Haloarcula hispanica*, which is the isolation host for three of them [37,38,41,53]. The one exception, HCIV-1, was isolated on *Haloarcula californiae*, and its plating efficiency is significantly lower with *Har. hispanica* than with the original host [52]. HCIV-1 can also infect *Haloarcula japonica* and *Halorubrum* sp. SS7-4 [52], and consequently the breadth of its host range is comparable to that of other viruses in the genus. SH1 infects *Halorubrum* CSW 2.09.4 and *Haloarcula* sp. PV7 strains, which are also host strains for PH1 and HHIV-2, respectively [38,41]. Transfection experiments have shown that some *Natrialba* and *Haloferax* strains, for example, support SH1 and PH1 replication and progeny virus production [41,56], which suggests that these viruses could have broader host ranges than observed under laboratory conditions.

Most of the investigated haloarchaeal viruses bind very slowly to their host receptors. Their adsorption rate constants range from ~10^−10^ to 10^−13^ mL/min, in contrast to bacteriophages that have adsorption rate constants of ~10^−9^ to 10^−10^ mL/min [57]. Adsorption rate constants of HCIV-1, SH1, and HHIV-2 fall into the same range as those of the other studied haloarchaeal viruses, being 5.7 × 10^−11^, 1.1 × 10^−11^, and 3.7 × 10^−12^ mL/min, respectively [38,42]. By 2 h post-infection (p.i.), ~50%, ~60%, and ~80% of HCIV-1, SH1, and HHIV-2 virus particles, respectively, are bound to the host cells [38,42]. Nothing is yet known about the receptors used by haloviruses. Electron micrographs taken during the early stages of HCIV-1 infection revealed an interesting phenomenon: tube-like formations between virus particles and the cell surface [42]. Similar structures are used as a genome delivery device by the icosahedral membrane-containing bacteriophage PRD1 and other viruses in the family *Tectiviridae* [58], thus prompting speculation that HCIV-1 might do likewise.

Alphasphaerolipoviruses are virulent and progeny viruses are released by host cell lysis. In single-step infection experiments, a concurrent drop in culture turbidity and release of progeny viruses is observed at 5–6 h, ~4 h, and ~12 h p.i. in SH1, HHIV-2, and HCIV-1, respectively [37,38,42]. By contrast, progeny PH1 virus release begins at ~6 h p.i. and continues, but turbidity does not start to decrease until ~15 h p.i. [41]. If PH1 adsorbs as slowly as the other haloarchaeal viruses [38,42,57,59], this observation could be explained by nonsynchronous virus release, which eventually results in cell lysis.

In addition to classical one-step infection experiments, the life cycles of SH1 and HCIV-1 have also been studied by real-time electrochemical and luminometric monitoring of cell integrity during virus exit [42,59]. Here, the measurement of the binding of the lipophilic anion phenyldicarbaundecaborane (PCB^−^) to exposed lipid bilayers [60], the level of dissolved oxygen in the medium, and intracellular ATP concentration assess the physiological state of the cell. Following infection by either SH1 or HCIV-1, cell integrity is compromised at the time of virus release. Concomitantly, haloarchaeal cell respiratory activity decreases and ATP leaks out of the cells [42,59]. As shown by transmission electron microscopy for SH1 and HHIV-2, empty and filled virus particles are observed inside the cells in the middle of the virus life cycle, which suggests that these viruses package their DNA into preformed procapsids [37,38]. Host cell lysis and increased cellular debris are observed later in the infection [37,38,42].

## 4. Synteny among the Genomes of HCIV-1, SH1, PH1 and HHIV-2

The ~30 kb genomes of alphasphaerolipoviruses are linear, dsDNA chromosomes with inverted terminal repeats and terminal proteins attached [35,38,41,42]. These properties imply that these viruses use protein-primed DNA replication [55], but a canonical DNA polymerase gene like that found in bacteriophage Φ29 [61] has not been identified in their genomes. Alphasphaerolipovirus genomes share over 56% nucleotide identity (Table 2); the most similar pair, SH1 and PH1, share 75.5% identity.

**Figure 2:**
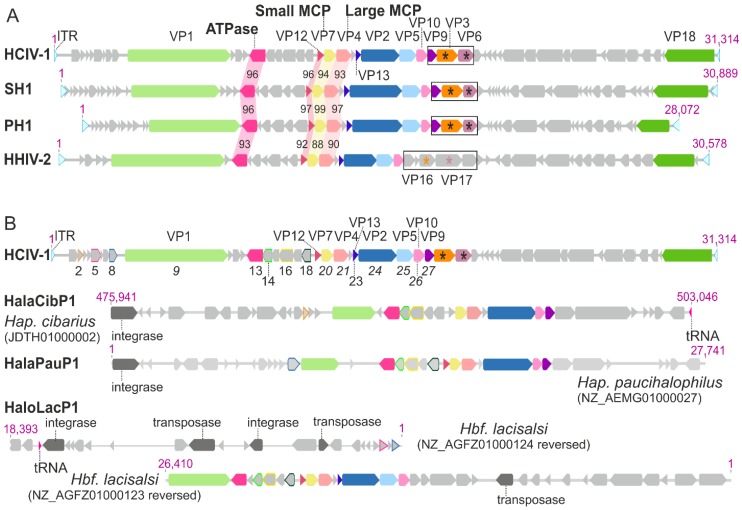
(**A**) Genomes of alphasphaerolipoviruses and (**B**) comparison of HCIV-1 genome with related putative proviral regions in archaeal chromosomes. (**A**,**B**) Grey arrows: open reading frames (ORFs)/genes. Coloured arrows: homologous genes coding for structural proteins. Light blue arrows: inverted terminal repeats (ITR). Virus ORFs or gene products are noted above the coding regions. The nomenclature for the virion proteins (VPs) is consistent in all four viruses except that the putative spike proteins (marked with an asterisk) of HHIV-2 (VP16 and VP17) correspond to VP3 and VP6 in the other viruses. The genomic regions including the genes encoding putative spike complex proteins are bordered with black lines. (**A**) Pairwise amino acid similarities (%) between the major membrane proteins (VP12), major capsid proteins (MCPs), or putative packaging ATPases are shown in between the genomes; (**B**) Designated ORFs/genes: HCIV-1 ORFs/genes with similarity to putative proviral sequences. Coloured arrows and coloured frames: similar structural proteins and putative proteins, respectively. Integrase and transposase encoding genes, as well as tRNA genes, are indicated in the putative proviruses.

Alphasphaerolipovirus genomes contain about 50 predicted open reading frames (ORFs) or genes that are arranged in a conserved order (Figure 2A). Some ORFs are found in all four genomes, others in as few as one. Nomenclature of viral structural proteins (VPs) is consistent for all four viruses and follows that established for SH1 [35]. The most conserved genes (or ORFs) are those that encode the small and large major MCPs (VP7 and VP4), the major membrane protein (VP12), and a putative packaging ATPase (Figure 2A). The least conserved of these genes (or ORFs) are those encoding the proteins of the vertex complex. Homologous genes encode the spike complex proteins of SH1, PH1, and HCIV-1. The HHIV-2 genes coding for the spike-associated host receptor recognition complex are sharply distinct from those in the other alphasphaerolipoviruses, as is the structure of their spikes (see below).

The abundance of proviral sequence elements related to alphasphaerolipoviruses in the chromosomes of halophilic archaea suggests that these viruses are more ubiquitous in saline environments than previously concluded. Putative proviruses have been identified in the chromosomes of *Haladaptus cibarius* (whole genome sequence; provirus HalaCibP1), *Haladaptus paucihalophilus* (draft genome; provirus HalaPauP1), and *Halobiforma lacisalsi* (draft genome; provirus HaloLacP1) (Figure 2B) [38,41,42,43,44]. Alphasphaerolipovirus-related sequences in these putative proviruses include ORFs coding for a putative packaging ATPase and for MCPs. ATPase and MCPs sequences are less conserved between proviral and viral ORFs (~65%–76% amino acid similarity) than among the four viruses. In the putative provirus HalaCibP1, alphasphaerolipovirus-related ORFs are located between an integrase gene and a tRNA gene. An integrase-encoding gene is also found in the putative provirus HalaPauP1 (*Hap. paucihalophilus* contig No. 27), while the adjacent contig (No. 28) contains a tRNA gene. In *Halobiforma lacisalsi*, the alphasphaerolipovirus-like provirus HaloLacP1 is located in contigs Nos. 123 and 124 where it is flanked by a transposase gene on contig No. 123 and a tRNA gene, two integrase genes, and two transposase genes on contig No. 124. It remains to be seen whether these putative proviruses are active and functional, or whether they represent proviral remnants.

## 5. Virion Organization of Alphasphaerolipoviruses

**Figure 3:**
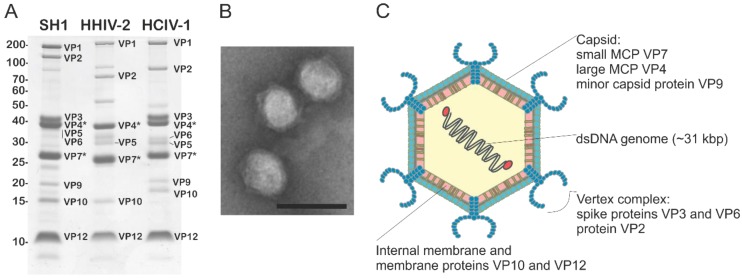
(**A**) Protein profiles of highly purified SH1, HHIV-2, and HCIV-1 virions with structural proteins designated. Asterisks: MCPs. Left column: molecular mass marker (kDa); (**B**) Electron micrograph of highly purified HCIV-1 particles stained with 3% (w/v) uranyl acetate. Scale bar: 100 nm; (**C**) Schematic model of HCIV-1 virion.

SH1, HHIV-2, and HCIV-1 assemble icosahedral virions, ~80 nm in diameter, that contain a dozen or more structural proteins (Figure 3A) [35,38,41,42]. The PH1 virion is reported to be smaller (~50 nm) [41]. Specific functions and locations were assigned to several of these structural proteins based on biochemical quantitative dissociation data and cryo-electron microscopy (cryo-EM) structural analysis [36,39,51]. For all four viruses, the icosahedral protein shell is decorated by spike complexes at the five-fold symmetry positions and encloses an internal lipid bilayer (see Figure 3B,C).

Spike complexes most likely function as host recognition devices, but the host range of these four viruses does not correlate with the observed spike complex shapes. The SH1 spikes are horn-shaped complexes with two-fold symmetry [36]. Sequence similarities suggest that PH1 and HCIV-1 have comparable, SH1-like spike structures. Although SH1, PH1, and HHIV-2 infect the same host species, HHIV-2 possesses a distinctly different host recognition complex. Cryo-EM observations of HHIV-2 virions reveal five-fold symmetric propeller-like objects with a fiber stemming from the center of the complex [39]. The vertex complex protein VP2 and large vertex structural protein VP1 were found in all four viruses. Proteins VP3 and VP6 are present in the complexes of SH1, PH1, and HCIV-1 [36,38,39,41,42,51]. The minor capsid protein VP9 is conserved in SH1, HHIV-2 and HCIV-1, but is not present in HHIV-2. Lastly, the complexes in HHIV-2 contain two proteins, VP16 and VP17, that do not show sequence similarity to any SH1 structural protein.

The internal viral membrane is composed of virus-encoded proteins and host lipids, the latter selectively acquired from the host lipid pool during virus assembly [35,39,42]. In the virion, the membrane closely underlies the capsid and takes on its shape. The apparent ordered density between the membrane and the overlying capsid that is revealed by cryo-EM might correspond to protein–membrane interactions that guided the assembly [36,39]. The two major membrane-associated proteins, VP10 and VP12, are conserved (57%-83% and 92%-97% amino acid similarity, respectively) among the alphasphaerolipoviruses and could serve as a scaffold for the capsid lattice. The major phospholipid species present in the virions of SH1, HHIV-2, and HCIV-1 are phosphatidylglycerol, phosphatidylglycerophosphate methyl ester, and phosphatidylglycerosulfate [35,39,42].

Cryo-EM and image reconstructions of SH1 and HHIV-2 virions revealed that their capsids are arranged in a pseudo *T* = 28 dextro lattice composed of MCPs VP4 and VP7 [36,39]. The only other known virus that has the same lattice symmetry is *Thermus* bacteriophage P23-77 in the genus *Gammasphaerolipovirus* [49]. Among the four alphasphaerolipoviruses, the small MCPs (VP7) share 86%–99% amino acid similarity, whereas the large MCPs (VP4) are even more conserved, having 90%–97% amino acid similarity. In SH1 and HHIV-2, MCPs VP4 and VP7 form pseudo-hexameric capsomers with either two or three towers [36,39]. VP7 is most probably a single β-barrel protein; VP4 most probably stacks two single β-barrel domains on top of each other, with the upper one forming a tower. An assembly model has been proposed for the HHIV-2 capsid [39], and the other alphasphaerolipoviruses likely mimic it. The capsid lattice is assembled from VP4-VP4 homodimers and VP4-VP7 heterodimers. The stability of the HHIV-2 capsid is compromised in a reducing environment, suggesting that disulphide bridges stabilize the protein-protein interactions in the capsid [39]. Conserved cysteine residues are found in both MCPs in SH1, PH1, and HHIV-2, but in only the small MCP VP7 in HCIV-1. The absence of such residues in the VP4 of HCIV-1 suggests a role for other capsid stabilization mechanisms, such as the observed membrane–capsid interactions.

## 6. Phylogenic Analysis and Structural Clustering of the Virion Core Elements of Sphaerolipoviruses

**Figure 4:**
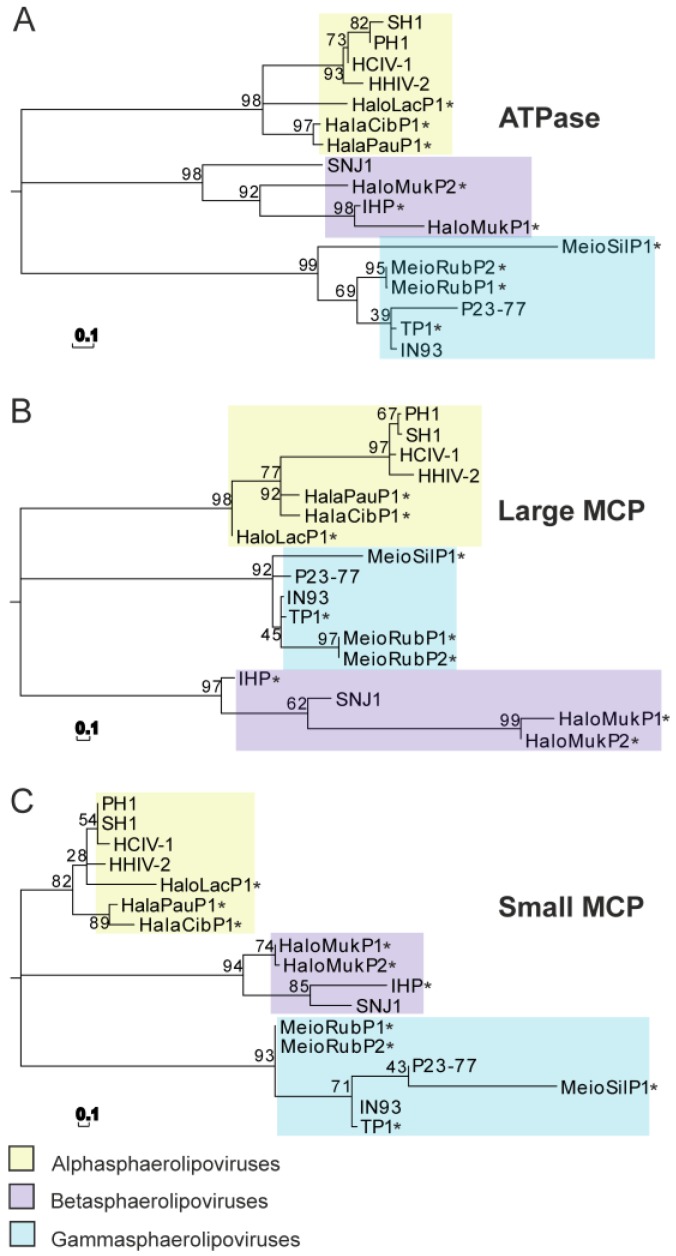
Maximum likelihood phylogenetic trees derived from protein sequences of the members of the family *Sphaerolipoviridae* and their related proviruses. (**A**) Packaging ATPases; (**B**) Large MCPs. (**C**) Small MCPs. Bars (0.1): the inferred number of substitutions per site. Reproduced with permission from [42] © American Society for Microbiology, mBio, 2016, vol. 7, no.4, e00699-16, doi:10.1128/mBio.00699-16.

Sequence-based phylogenetic analysis of the most conserved virion proteins—two MCPs and a packaging ATPase—revealed that seven sphaerolipoviruses and their related proviruses cluster into three distinct clades that correspond to the three genera in the family (Figure 4) [42].

**Figure 5:**
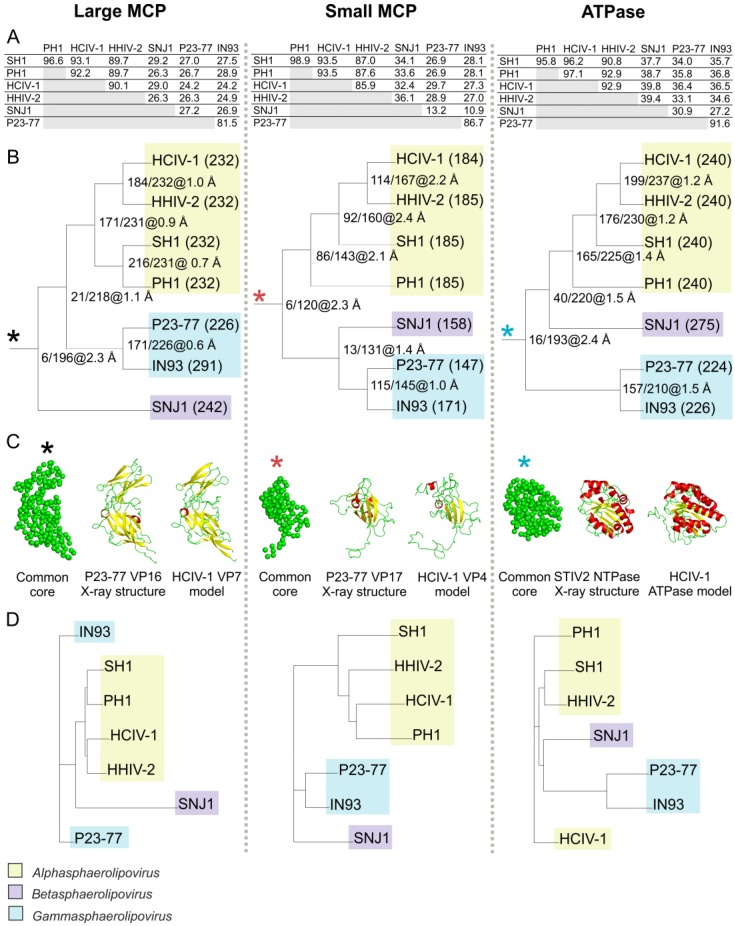
Structural phylogeny based on the predicted structural models of the large MCPs (left), small MCPs (middle), and putative packaging ATPases (right) of the viruses in the family *Sphaerolipoviridae*. (**A**) Amino acid similarities (%) between proteins calculated with EMBOSS Needle [64]; (**B**) The clustering based on structural models generated by Iterative Threading ASSEmbly Refinement (I-TASSER) sequence-based predictions [62]. Each protein was compared against the corresponding common core (asterisks). Brackets: the number of amino acid residues used in comparisons. Tree node labels: MMM/NNN@X.Y Å, where MMM = number of identical residues at equivalent positions, NNN = total number of equivalent residues in the common core for all proteins in the subtree below that node, and X.Y Å = average root-mean-square deviation (rmsd) of atomic positions over all equivalent (structurally aligned) residues; (**C**) The average α-carbon positions of the common cores (asterisks) with equivalent residues depicted in green; the initial structural templates used: X-ray structures of *Thermus* phage P23-77 VP17 (PDB ID: 3ZMN) and VP16 (PDB ID: 3ZN4), and *Sulfolobus* virus STIV2 NTPase (PDB ID: 4KFR); models of the HCIV-1 MCPs (VP7 and VP4) and putative packaging ATPase, as examples. Yellow: β-sheets; red: α-helices; green: loops. Visualized with the PyMOL Molecular Graphics System, Version 1.8 Schrödinger, LLC; (**D**) Phylogenic trees of the protein models based on their common cores and including only equivalent residues (Homologous Structure Finder (HSF) Program) [63]. Branch lengths are calculated as in [63]. The trees were visualized with Dendroscope [65]. For methodological details, see [63].

The same clustering is observed when the MCPs and packaging ATPases are structurally compared among the same viruses (Figure 5). This comparison used the high resolution structures of the P23-77 large and small MCPs (VP16 and VP17) [49] together with structural models of other proteins generated by the Iterative Threading ASSEmbly Refinement (I-TASSER) web server [62]. Since the amino acid sequence similarities are sometimes low within this group of viruses (Figure 5A), each protein structural model was predicted separately. The large and small MCPs were all modeled using the structures of *Thermus* phage P23-77 MCPs VP16 and VP17, respectively, as the initial templates (Protein Data Bank (PDB) ID: 3ZN4 and 3ZMN) [49]. Similarly, templates for the putative packaging ATPases were (i) for HCIV-1 and PH1: *Escherichia coli* FtsK motor domain (PDB ID: 2IUS); (ii) for HHIV-2 and SH1: HerA ATPase (PDB ID: 4D2I); and (iii) for IN93, SNJ1 and P23-77: the putative packaging ATPase of *Sulfolobus* virus STIV2 (PDB ID: 4KFR).

All virus protein models used possessed good C-scores (a confidence score for estimating the quality of predicted models) and TM-scores (a parameter for structural similarity of two protein models), as well as good coverage. We identified a structural core composed of 196 amino acid residues common to all large MCPs. The 2.3 Å average root-mean-square deviation (rmsd) (Figure 5B,C, left) demonstrated that these MCPs share the same protein fold, a fold that is characterized by two single β-barrels stacked one on top of the other. The structural model of HCIV-1 VP7 is compared with the structures of P23-77 VP16 and the common core in Figure 5C (left).

A similar investigation found a total of 120 residues common to all sphaerolipovirus small MCPs. Their average rmsd of 2.3 Å (Figure 5B, middle) indicated that they are all single β-barrel proteins with a fold similar to that of P23-77 VP17 [49]. Turning to the putative packaging ATPases, we found that, although the initial templates differed, the final models were extremely similar and shared a common core of 193 residues (Figure 5B,C, right). Overall, all three of the identified common cores shared 82%–87% equivalent residues (see next paragraph) when compared to the shortest protein used in the comparison. In all three cases, that protein was the one from P23-77.

The structural models and the available structures were also clustered using the Homologous Structure Finder (HSF) (Figure 5D) [63]. When given a set of protein structures and models, HSF identifies a structural core of equivalent amino acid residues among them based on similarities of amino acid geometry, secondary structure, and physicochemical properties. This method of structure-based clustering yielded results that paralleled the previously established sequence-based classification (Figure 4 and Figure 5D). In addition, in these HSF analyses, the structural model closest to that of HCIV-1 was always that of HHIV-2.

## 7. Conclusions

**Figure 6:**
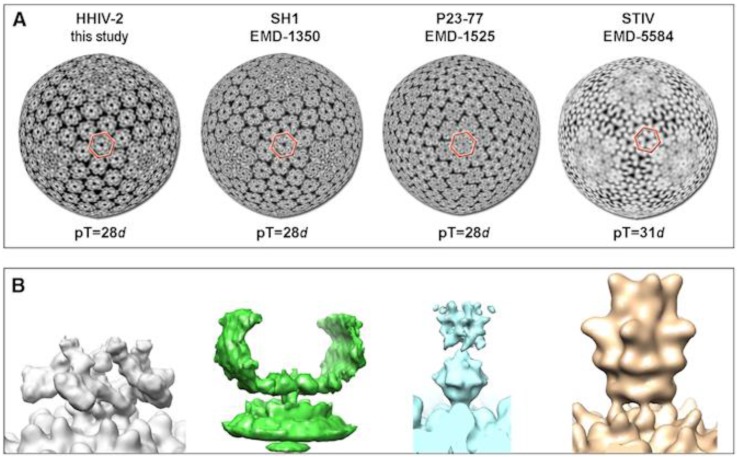
Icosahedral membrane-containing viruses with conserved capsid elements but highly divergent host recognition structures. (**A**) Radial cross sections at the base of the conserved pseudo-hexameric capsomers (red indicates the base of one capsomer). The capsomers of HHIV-2, SH1, and P23-77 are composed of six vertical single β-barrel MCPs, whereas STIV represents an example that uses instead three vertical double β-barrel MCPs. EMDataBank accession numbers are provided. HHIV-2 is deposited under the accession number of EMD-3109 (“this study” here refers to [39]); (**B**) The five-fold vertex complexes of these four viruses (not to scale). Reproduced with permission from [39] Reprinted from Structure, vol. 23, no. 10, David Gil-Carton, Salla T. Jaakkola, Diego Charro, Bibiana Peralta, Daniel Castaño-Díez, Hanna M. Oksanen, Dennis H. Bamford, and Nicola G.A. Abrescia, Insight into the assembly of viruses with vertical single β-barrel major capsid proteins, Pages No. 1866-1877, Copyright 2015, with permission from Elsevier. doi: 10.1016/j.str.2015.07.015.

Currently the genus *Alphasphaerolipovirus* of the family *Sphaerolipoviridae* comprises four halophilic archaeal viruses—SH1, HHIV-2, PH1, and HCIV-1—that were isolated from different hypersaline environments. All four assemble tailless, icosahedral virions that contain an internal membrane (Figure 1). Despite their widely-separated sites of origin, these four possess a high level of sequence similarity, especially in their core structural and assembly proteins, i.e., the two MCPs and a packaging ATPase (Figure 4 and Figure 5). A pseudo-hexameric capsid lattice arranged in *T* = 28 symmetry is common to alphasphaerolipoviruses SH1 and HHIV-2 and the gammasphaerolipovirus P23-77 (Figure 6A) [36,39,45]. The MCPs of alpha- and gammasphaerolipoviruses share low sequence similarities (24%–29%; Figure 5A), but the predicted folding of their MCPs (Figure 5C) and their overall virion architecture are highly conserved. By contrast, the vertex complexes, most probably involved in host recognition, may differ significantly (Figure 6B).

From a structural perspective, the virion architecture of the sphaerolipoviruses places them within the PRD1-adenovirus structural lineage [30]. This lineage comprises viruses with a pseudo-hexameric virion lattice built of either (*i*) one type of MCP with two vertical β-barrel domains, or (*ii*) two MCP species with single β-barrels [30,39]. The first subgroup is well-established and includes at least bacteriophages PM2 (*Corticoviridae*) and PRD1 (*Tectiviridae*), archaeal viruses STIV and STIV2 (*Turriviridae*), as well as eukaryotic viruses PBCV-1 (*Phycodnaviridae*), vaccinia virus (transition state in particle maturation; *Poxviridae*), human adenovirus (*Adenoviridae*), and virophage Sputnik (*Lavidaviridae*) [11,13,21,22,23,24,25,26]. The second subgroup currently includes sphaerolipoviruses together with halophilic bacterial virus *Salisaeta* icosahedral phage 1 (SSIP-1; unclassified) [66]. Thus, all of the above-mentioned viruses that are currently classified in at least eight viral families can be grouped into one structural lineage [30,31]. It will be intriguing to see how far this structural conservation extends and what novel virus morphotypes may await discovery in extreme environments.

## Figures and Tables

**Table 1:** Virus species belonging to the three genera of the family *Sphaerolipoviridae*.

Genus	Virus species	Virus isolate	Isolation source	Isolation host	Life cycle	Virion diameter (nm)	*T* number ^2^	Internal membrane ^3^	dsDNA genome ^4^	Reference
*Alpha-sphaero-lipovirus*	*Haloarcula hispanica virus SH1* (type species)	SH1	Saline lake, Australia	*Haloarcula hispanica*	Lytic	~80	*T* = 28, dextro	Yes	Linear 30,889 bp AY950802	[35,36,37]
	*Haloarcula hispanica virus HHIV2*	HHIV-2	Solar saltern, Italy	*Haloarcula hispanica*	Lytic	~80	*T* = 28, dextro	Yes	Linear 30,578 bp JN968479	[38,39]
	*Haloarcula hispanica virus PH1*	PH1	Saline lake, Australia	*Haloarcula hispanica*	Lytic	~50	nd	nd	Linear 28,072 bp KC252997	[41]
	*Haloarcula virus HCIV1* ^1^	HCIV-1	Solar saltern, Thailand	*Haloarcula californiae*	Lytic	~70	nd	Yes	Linear 31,314 bp KT809302	[42,52]
*Beta-sphaero-lipovirus*	*Natrinema virus SNJ1* (type species)	SNJ1	Salt mine (*Natrinema* sp. J7-1)	*Natrinema* sp. J7-2	Lysogenic	~70–75	nd	Yes	Circular 16,341 bp AY048850.1	[7,40]
*Gamma-sphaero-lipovirus*	*Thermus virus P23-77* (type species)	P23-77	Alkaline hot spring, New Zealand	*Thermus thermophilus*	Lytic	~78	*T* = 28, dextro	Yes	Circular 17,036 bp GQ403789	[45,46,49]
	*Thermus virus IN93*	IN93	Hot spring soil, Japan (*Thermus aquaticus* TZ2)	*Thermus thermophilus*	Lysogenic	~130	nd	nd	Circular 19,604 bp AB063393	[47,48]

^1^ Proposed; ^2^ *T*, triangulation number; nd, not determined; ^3^ Lipids have been demonstrated to be a structural component of the virion; ^4^ Genome type and size and GenBank accession number. HHIV-2: *Haloarcula hispanica* icosahedral virus 2; HCIV-1: *Haloarcula californiae* icosahedral virus 1.

**Table 2:** Overall nucleotide identities (%) between virus genomes.

	SH1	PH1	HHIV-2
**HCIV-1**	63.0	58.1	56.5
**SH1**	100	75.5	57.9
**PH1**	75.5	100	56.4
**HHIV-2**	57.9	56.4	100
